# Sex differences in personality disorders in a Chinese clinical population

**DOI:** 10.3389/fpsyt.2022.1006740

**Published:** 2022-09-23

**Authors:** HongZhen He, Mei Xu, ZhangYing Fei, Yuou Xie, XinYi Gu, HongLiang Zhu, JunJie Wang

**Affiliations:** ^1^Department of Clinical Psychology, The First Affiliated Hospital of Fujian Medical University, Fuzhou, China; ^2^Institute of Mental Health, Suzhou Guangji Hospital, The Affiliated Guangji Hospital of Soochow University, Suzhou, China; ^3^Wujiang Mental Rehabilitation Hospital, Suzhou, China; ^4^Department of Clinical Medicine, The First Clinical Medical College of Nanjing Medical University, Nanjing, China; ^5^Department of Medical Information Management, Wenzhou Medical University, Wenzhou, China

**Keywords:** personality disorder (PD), sex, clinical, outpatients, China

## Abstract

**Introduction:**

Sex differences in the frequency and severity of personality disorders (PDs) have been widely reported in Western countries. However, limited literature suggests a similar sex distribution in the Chinese clinical population. This study investigated sex differences in self-reported and interviewed patients with PDs in a clinical population in China.

**Materials and methods:**

The participants were 1,389 consecutive outpatients with a mean age of 30.5 years, including 634 (45.6%) males and 755 (54.4%) females. Self-reported PD traits were assessed using the Personality Diagnostic Questionnaire Fourth Edition Plus (PDQ-4+). PDs were diagnosed according to the Structured Clinical Interview for Diagnostic and Statistical Manual of Mental Disorders, Fourth Edition (DSM-IV) Axis II (SCID-II).

**Results:**

Male outpatients reported more paranoid, schizotypal, antisocial, and passive-aggressive PD traits, whereas females reported more borderline PD traits on the PDQ-4+. Self-reported PD traits in male outpatients were more likely to reach the positive threshold of antisocial PD than in females (χ^2^ = 5.293, *p* = 0.021). Males were more likely to meet the criteria for schizoid (χ^2^ = 5.050, *p* = 0.025), narcissistic (χ^2^ = 27.244, *p* < 0.001), antisocial (χ^2^ = 11.430, *p* = 0.001), avoidant (χ^2^ = 5.098, *p* = 0.024), and obsessive-compulsive PD (χ^2^ = 5.496, *p* = 0.019) diagnoses in the SCID-II. In contrast, females were more likely to meet the criteria of histrionic (χ^2^ = 12.327, *p* = 0.001), borderline (χ^2^ = 28.538, *p* < 0.001), and dependent (χ^2^ = 4.919, *p* = 0.027) diagnoses.

**Discussion:**

These findings indicate gender differences in the traits, frequency, and pattern of PDs when assessed in a Chinese clinical population.

## Introduction

Personality disorder (PD) is increasingly recognized as an important clinical diagnosis in psychiatry, marked by significant and enduring inflexible and inadequate inner experiences and behaviors (1). Although important in psychiatric practice and closely related to comorbidity, outcomes, and treatment effects of other mental disorders, PD is often overlooked in clinical practice. Notably, its diagnosis is ignored when based on the Chinese psychiatric diagnostic system (2). Even basic questions, such as the prevalence of PD in clinical populations, have not been well studied. A question of increasing interest (3–5) within epidemiological research is whether there are differences in specific PDs between sexes.

In the general population, females are generally perceived as more emotional and neurotic, whereas males are associated with more assertive behavior (6). The findings of Paris (7) suggested that sex differences in personality traits in the general population might be reflected in the sex ratio of PDs in clinical patients. From clinical experience, some PDs, such as borderline, histrionic, and dependent, are more commonly diagnosed in females and other PDs, such as antisocial and paranoid, are historically seen as “typically male.”

Previous studies (8–10) evaluated sex differences in PDs in general and clinical populations. However, limited data are available regarding the possible role of sex in the assessment process using self-report and clinical interview methods in a Chinese clinical population. This study addressed this issue by examining sex differences in self-reported PD traits and interviewing PD diagnoses in a clinical population. The primary goal was to determine whether self-reported PD traits and interview-based PD diagnoses were consistent between the sexes.

## Methods

### Study design and sample

This was a cross-sectional study of self-report and structured interview methods in patients at the First Affiliated Hospital of Fujian Medical University. To evaluate the sex distribution of PDs, this study enrolled consecutive outpatients seeking mental health services between 2016 and 2022. It was performed according to the regulations on the use of human subjects established by the Ethics Committee of First Affiliated Hospital of Fujian Medical University. The inclusion criteria for entering the screening process were: (i) age between 18 and 70 years; (ii) capacity to provide informed consent; (iii) completion of at least 6 years of primary education; (iv) willingness to understand their personality problems; (v) stable treatment condition. The exclusion criteria were: (i) severe somatic diseases such as pneumonia, cancer, and heart failure; (ii) intellectual disability.

After informed consent was obtained, participants were screened for PD traits using a self-report questionnaire, the Personality Diagnostic Questionnaire Fourth Edition Plus (PDQ-4+). After screening, senior psychiatrists used the Structured Clinical Interview for Diagnostic and Statistical Manual of Mental Disorders, Fourth Edition (DSM-IV) (11). Axis II (SCID-II) for PD diagnosis. The clinical diagnoses of all participants were made by their doctors according to routine clinical practice, which was mainly based on codes from the International Classification of Diseases, version 10 (ICD-10). The diagnostic categories used in this analysis were: (1) psychosis; (2) mood disorders; (3) anxiety disorder; (4) others.

### PD assessments

#### Self-report PD traits

The PDQ-4+ (12) is a self-report questionnaire used to evaluate pathological personality traits based on the DSM-IV (11) criteria. This questionnaire screens 12 types of PDs, including paranoid, schizoid, schizotypal, histrionic, narcissistic, borderline, antisocial, avoidant, dependent, obsessive-compulsive, depressive, and passive-aggressive. The Chinese version of the PDQ-4+ has been validated to have high sensitivity (0.89) and moderate specificity (0.65) for screening PD patients (2). It has been widely used for PD assessments in China (2, 13–17). The PDQ-4+ comprises 107 true-false items and “yes” indicates a pathological reaction. Higher subscale scores indicate a greater likelihood of having a certain type of PD. A subscale score of more than three points indicates traits of a specific PD.

#### Structured interview PD diagnosis

SCID-II (18, 19) is a semi-structured diagnostic interview for clinicians and researchers designed to evaluate DSM-IV (11) personality disorders across clusters A, B, and C. It contains 119 items in a yes/no response format. The Chinese version of SCID-II was translated and implemented in this study (17). Previous studies have demonstrated that the Chinese version of SCID-II = has a relatively high test-retest reliability of 0.70, with a median coefficient for internal consistency of 0.70, which is highly consistent (0.90) with clinical diagnoses.

### Data analysis

Data were analyzed using SPSS version 16.0 (SPSS Inc., Chicago, IL, USA) and independently entered into the database twice. Data checking and cleaning were performed before the analysis, considering range and consistency. Frequencies and percentages were calculated for qualitative variables such as sex and marital status. Means (M) and standard deviations (SD) were calculated for quantitative variables such as age. The difference between the means and proportions was evaluated using an independent *t*-test and chi-square test, respectively. The mean PDQ-4+ subscale scores and percentages of SCID-II PD diagnoses were transferred to a spreadsheet program (Excel, Microsoft, USA). Statistical significance was set at *p* < 0.05.

## Results

The demographic and clinical characteristics of male and female outpatients (*N* = 1,389) are given in Table 1. No differences were found regarding age, occupation, education, and family history, between the two groups. Compared with females, a greater proportion of male outpatients were single, showed introverted personality characteristics, and were diagnosed with psychosis; a lesser proportion had religious beliefs, a lower educational level, and been diagnosed with mood and anxiety disorders.

**Table 1:** Demographic and clinical characteristics in male and female outpatients.

**Variables**	**Total**	**Male**	**Female**	**Comparison**	
				***t/χ^2^***	***p-*value**
Cases [*n* (%)]	1,389	634 (45.6)	755 (54.4)	-	-
Age (years) [*Mean (SD)*]	30.5 (9.6)	30.0 (9.6)	30.8 (9.6)	1.544	0.123
Age range (years)	18–60	18–60	18–60	-	-
Single	704 (50.7)	372 (58.7)	332 (44.0)	33.165	**< 0.001**
Married	596 (42.9)	236 (37.2)	360 (47.7)		
Divorced	58 (4.2)	17 (2.7)	41 (5.4)		
Widowed	31 (2.2)	9 (1.4)	22 (2.9)		
Unemployed [*n (%)*]	154 (11.1)	64 (10.1)	90 (11.9)	1.165	0.280
Student [*n (%)*]	295 (21.2)	145 (22.9)	150 (19.9)	1.858	0.173
Religious belief [*n (%)*]	302 (21.8)	118 (18.6)	185 (24.5)	7.013	**0.008**
**Education**					
Middle school [*n (%)*]	180 (13.0)	82 (12.9)	98 (13.0)	0.071	0.965
High school [*n (%)*]	407 (29.3)	188 (29.7)	219 (29.0)		
College or higher [*n (%)*]	802 (57.7)	364 (57.4)	438 (58.0)		
**Personality character [** ***n (%)*** **]**					
Introversion	456 (32.8)	267 (42.1)	189 (25.0)	51.138	**< 0.001**
In-between	695 (50.0)	289 (45.6)	406 (53.8)		
Extroversion	238 (17.1)	78 (12.3)	160 (21.2)		
Family history of first-degree relatives [*n* (%)]	88 (6.3)	40 (6.3)	48 (6.4)	0.001	0.530
**Diagnosis [*****n*** **(%)]**					
Psychosis	183 (13.2)	96 (15.1)	87 (11.5)	26.725	**< 0.001**
Mood disorders	444 (32.0)	160 (25.2)	284 (37.6)		
Anxiety disorder	406 (29.2)	211 (33.3)	195 (25.8)		
Others	356 (25.6)	167 (26.3)	189 (25.0)		

Significant in bold.

Table 2 shows the self-reported PD traits from PDQ-4+. Male outpatients reported more paranoid, schizotypal, antisocial, and passive-aggressive PD traits, while females reported more borderline PD traits.

**Table 2:** Self-reported PDs traits using PDQ-4+ compared between male and female outpatients.

**Variables**	**Total**	**Male**	**Female**	**Comparison**	
				***t***	***p***
Cases	1,389	634 (45.6)	755 (54.4)	-	-
Paranoid PD	2.9 (1.9)	3.0 (1.9)	2.7 (1.9)	2.586	**0.010**
Schizoid PD	2.4 (1.7)	2.4 (1.7)	2.3 (1.7)	0.965	0.335
Schizotypal PD	3.4 (2.2)	3.5 (2.2)	3.3 (2.2)	1.984	**0.047**
Histrionic PD	3.6 (1.8)	3.5 (1.9)	3.6 (1.8)	1.341	0.180
Narcissistic PD	3.3 (2.1)	3.4 (2.1)	3.3 (2.1)	0.748	0.454
Borderline PD	4.1 (2.4)	4.0 (2.4)	4.3 (2.5)	2.229	**0.026**
Antisocial PD	1.5 (1.6)	1.7 (1.6)	1.3 (1.6)	4.481	**< 0.001**
Avoidant PD	3.7 (2.1)	3.8 (2.1)	3.7 (2.1)	1.017	0.309
Dependent PD	3.0 (2.2)	3.0 (2.2)	3.1 (2.2)	0.776	0.438
Obsessive-compulsive PD	3.9 (2.0)	3.9 (2.0)	3.8 (2.0)	1.071	0.284
Passive-aggressive PD	2.8 (1.7)	2.9 (1.6)	2.7 (1.7)	2.285	**0.022**
Depressive PD	3.6 (2.1)	3.6 (2.1)	3.7 (2.1)	0.179	0.858
Total PDQ-4+ score	38.2 (17.0)	38.7 (16.9)	37.7 (17.0)	1.039	0.299

Significant in bold. The numbers represent the score of PDQ-4+ subscales.

**Figure 1:**
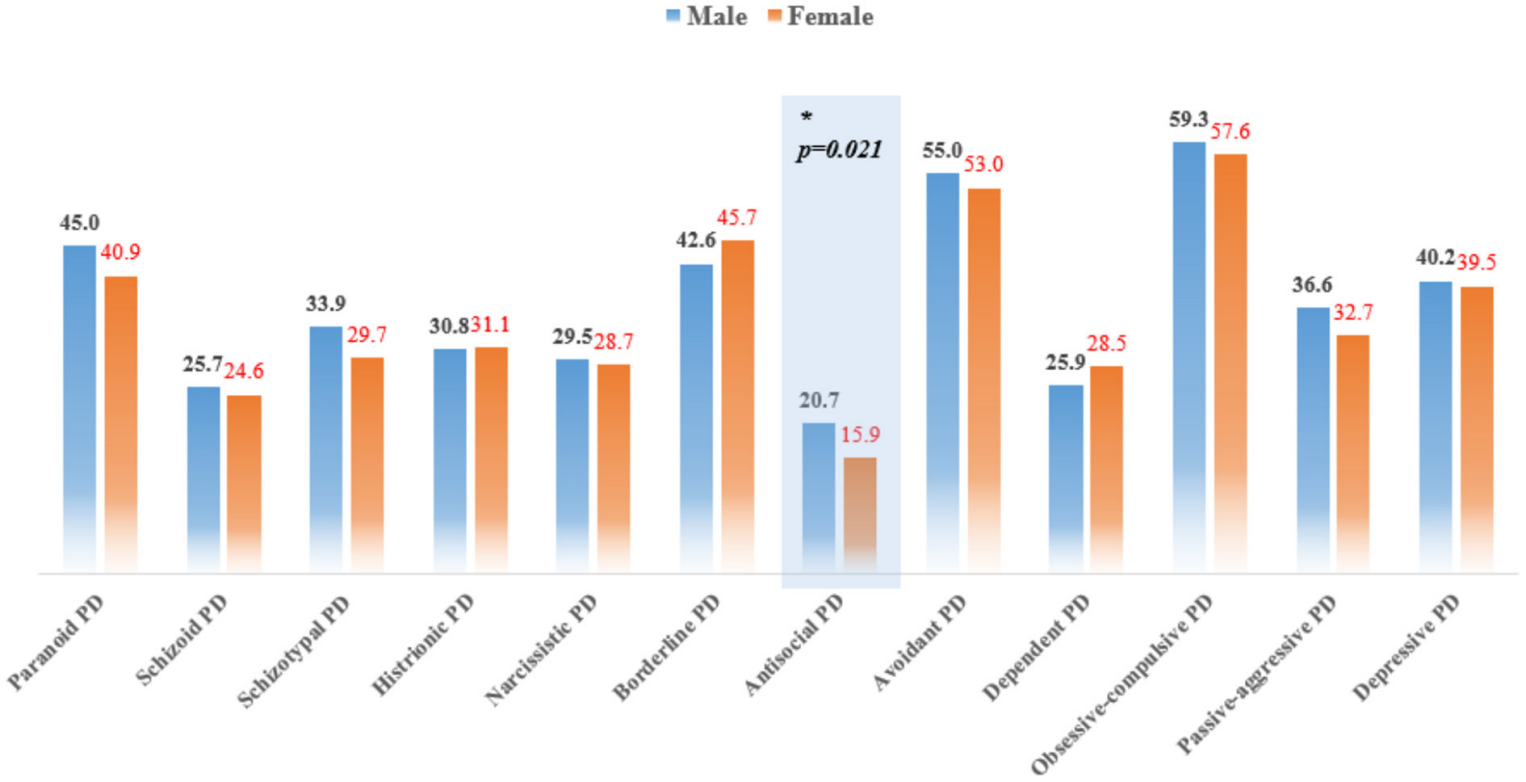
Frequency of PDQ-4+ self-reported PDs, compared between male and female outpatients. Comparisons were evaluated with the χ^2^-test.

The frequency rates calculated using PDQ-4 + are presented in Figure 1. Self-reported PD traits in male outpatients were more likely to reach the positive threshold of antisocial PD compared with female patients (χ^2^ = 5.293, *p* = 0.021).

**Figure 2:**
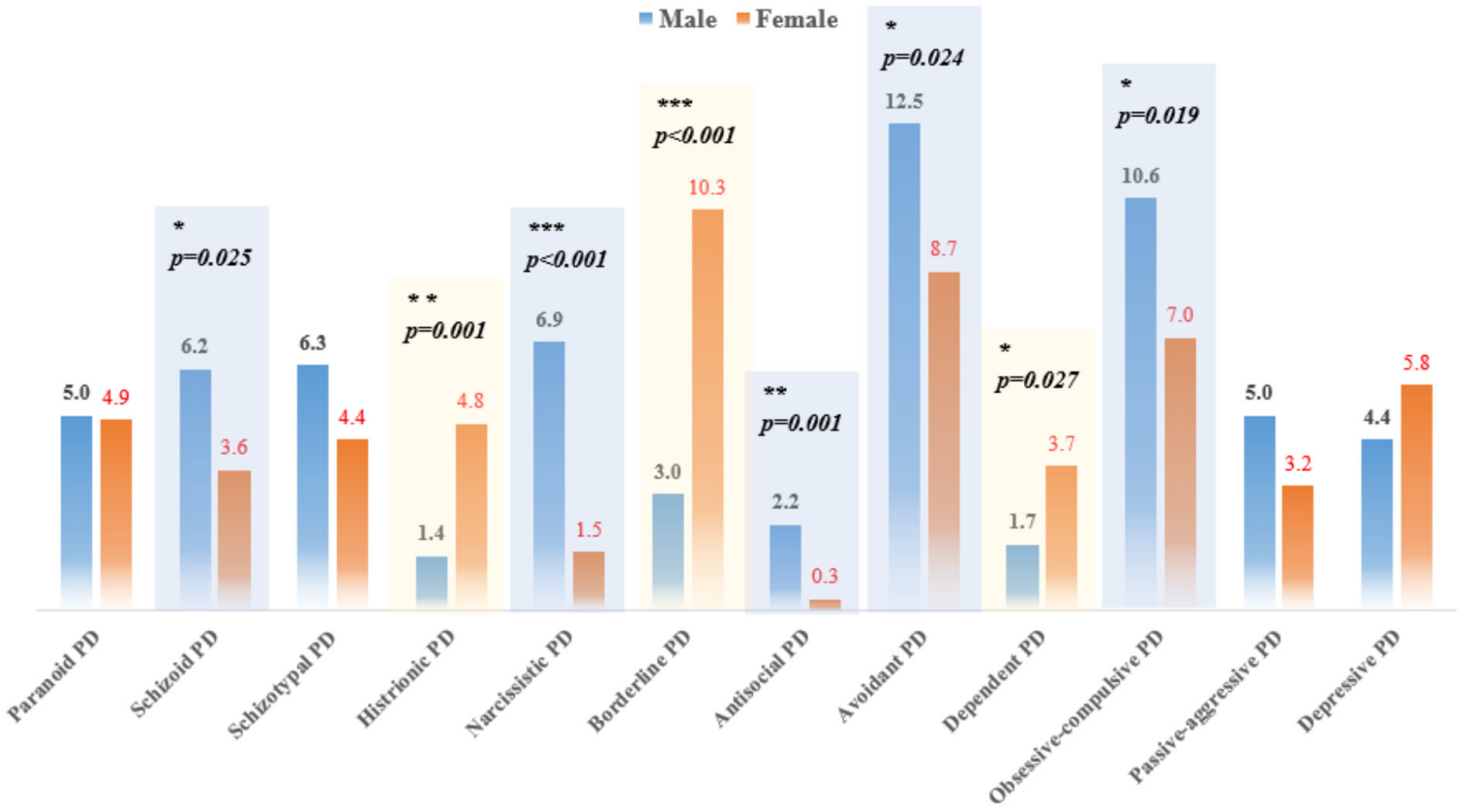
Prevalence of SCID-II diagnosed PDs among patients with psychotic and non-psychotic disorders.

According to the results of SCID-II, obsessive-compulsive (10.6%) and avoidant (12.5%) PDs were the most common among male outpatients, whereas borderline PD was the most common among female outpatients (prevalence rate > 10%). Males were more likely to meet the criteria of schizoid (χ^2^ = 5.050, *p* = 0.025), narcissistic (χ^2^ = 27.244, *p* < 0.001), antisocial (χ^2^ = 11.430, *p* = 0.001), avoidant (χ^2^ = 5.098, *p* = 0.024), and obsessive-compulsive PD (χ^2^ = 5.496, *p* = 0.019) diagnoses. In contrast, females were more likely to meet the criteria of histrionic (χ^2^ = 12.327, *p* = 0.001), borderline (χ^2^ = 28.538, *p* < 0.001), and dependent (χ^2^ = 4.919, *p* = 0.027) diagnoses (Figure 2).

## Discussion

### Key findings

To the best of our knowledge, this is the first study that utilized self-report and clinical interview methods to describe sex differences in PDs in a large representative Chinese population. Three key findings were noted. First, within the complete sample of this study, sex differences in self-reported PD traits in the total score of the PDQ-4+ were not significant. This suggests that differences in overall PD pathology in the entire population may be subtle. Second, in self-reported specific PD traits, females appeared to have more severe traits of borderline PD, while males showed more severe traits of narcissistic, antisocial, paranoid, passive-aggressive, and schizoid PDs. Third, in structured interview diagnoses, borderline, histrionic, and dependent PDs predominated in females, whereas schizoid, narcissistic, antisocial, avoidant, and obsessive-compulsive PDs predominated in males.

### Cluster A PDs

Although the reliability and validity of the diagnostic criteria for paranoid PD have been questioned repeatedly (20), it is one of the most common PDs in clinical practice. Consistent with findings from a large survey of the 2001–2002 National Epidemiologic Survey on Alcohol and Related Conditions (21), more males than females, in the clinical population, met paranoid PD criteria. Schizotypal PD is slightly more common in males according to the DSM, Fifth Edition (DSM-V) (22), which is consistent with our results. However, this finding is inconsistent with that of another study that reported that the lifetime prevalence of schizotypal PD was 4.2% in females and 3.9% in males (20). Sex-specific patterns of Axis I comorbidities, including psychosis and non-psychosis (23, 24), may lead to inconsistencies in different studies.

### Cluster B PDs

Borderline and histrionic PDs were predominately “female” PDs with a sex ratio of 3:1, which is highly consistent with the DSM-V (22). However, the prevalence of borderline PD reported no sex differences in the general population in a study by Torgersen et al. (25). One explanation for these opposing results is a selection bias in clinical samples that females with borderline PD may be more inclined to seek help from mental health services (26). Further analysis of the symptomatic characteristics of borderline PD was performed by Hoertel et al. (27), using applied methods based on the item response theory, to examine sex differences in the likelihood of reporting borderline PD symptoms. They found that females were more likely than males to experience suicidal/self-mutilation behavior, affective instability, and chronic feelings of emptiness.

Contrary to borderline and histrionic PD, narcissistic and antisocial PDs are predominately “male” PDs with a sex ratio of 4–7:1 in this study, thereby accounting for the most striking sex differences in PDs. These findings are consistent with other studies (28–31) which reported the lifetime prevalence of narcissistic and antisocial PDs to be higher in males. The theoretical explanation for sex differences is based on diverse developmental trajectories, wherein male childhood manifests more externalizing impairments, such as conduct disorder, while female childhood manifests more internalizing symptoms, such as anxiety and affective symptoms (32).

### Cluster C PDs

Obsessive-compulsive and avoidant PDs were reported to be higher in males, whereas dependent PD was more common in females. A similar finding was reported in the general population wherein the prevalence of dependent PD in females was 0.6 vs. 0.4% in males (21). This may be related to males' reluctance to express dependency characteristics (33). One study reported that the sex ratio of obsessive-compulsive PD is unbalanced, indicating its predominance among males. This fits the reported ratio in the DSM-V (22) with males affected twice as often as females. Avoidant PD was more prevalent in males in the structured interview, but there was no corresponding difference in the self-reported method. This is likely related to the deviation between patients and doctors in understanding avoidant PD traits.

### Limitations

Similar to other studies, this study has several limitations. First, the comorbid Axis-I disorders in male outpatients were significantly different from those in females (a greater proportion of diagnoses of psychosis but less in mood and anxiety disorders). Thus, there may be a potential selection bias, as comorbid Axis-I disorders are likely to impact PD assessments. Second, the data were cross-sectional, whereas cohort studies are more valuable for the validation of PD diagnosis. Third, this was a single-center study and our sample may not represent the entire Chinese population. Consequently, the generalizability of the findings is limited.

## Conclusion

Our results show that borderline, histrionic, and dependent PDs were more prominent in females. In contrast, narcissistic, antisocial, avoidant, and obsessive-compulsive PDs were more prominent in males. Although the cultural differences between China and the West are significant, the sex differences in specific PDs in China seem to be consistent with the findings of Western countries.

## Data availability statement

The raw data supporting the conclusions of this article will be made available by the authors, without undue reservation.

## Ethics statement

The studies involving human participants were reviewed and approved by Institutional Review Board of the First Affiliated Hospital of Fujian Medical University. The patients/participants provided their written informed consent to participate in this study.

## Author contributions

HH contributed to the data acquisition and writing of the first draft manuscript. MX and ZF managed statistical analyses. YX and XG managed literature searches. HZ and JW contributed to the experimental design and data interpretation. All authors contributed to the article and approved the submitted version.

## Conflict of interest

The authors declare that the research was conducted in the absence of any commercial or financial relationships that could be construed as a potential conflict of interest.

## Publisher's note

All claims expressed in this article are solely those of the authors and do not necessarily represent those of their affiliated organizations, or those of the publisher, the editors and the reviewers. Any product that may be evaluated in this article, or claim that may be made by its manufacturer, is not guaranteed or endorsed by the publisher.
